# Cross-talk of nitric oxide and reactive oxygen species in plant programed cell death

**DOI:** 10.3389/fpls.2013.00314

**Published:** 2013-08-16

**Authors:** Yiqin Wang, Gary J. Loake, Chengcai Chu

**Affiliations:** ^1^State Key Laboratory of Plant Genomics and National Center for Plant Gene Research (Beijing), Institute of Genetics and Developmental Biology, Chinese Academy of SciencesBeijing, People’s Republic of China; ^2^Institute of Molecular Plant Sciences, School of Biological Sciences, University of EdinburghEdinburgh, UK

**Keywords:** nitric oxide, reactive oxygen species, programed cell death, hypersensitive response, leaf senescence

## Abstract

In plants, programed cell death (PCD) is an important mechanism to regulate multiple aspects of growth and development, as well as to remove damaged or infected cells during responses to environmental stresses and pathogen attacks. Under biotic and abiotic stresses, plant cells exhibit a rapid synthesis of nitric oxide (NO) and a parallel accumulation of reactive oxygen species (ROS). Frequently, these responses trigger a PCD process leading to an intrinsic execution of plant cells. The accumulating evidence suggests that both NO and ROS play key roles in PCD. These redox active small molecules can trigger cell death either independently or synergistically. Here we summarize the recent progress on the cross-talk of NO and ROS signals in the hypersensitive response, leaf senescence, and other kinds of plant PCD caused by diverse cues.

## NO, REACTIVE NITROGEN SPECIES, AND PROTEIN *S*-NITROSYLATION

**FIGURE 1:**
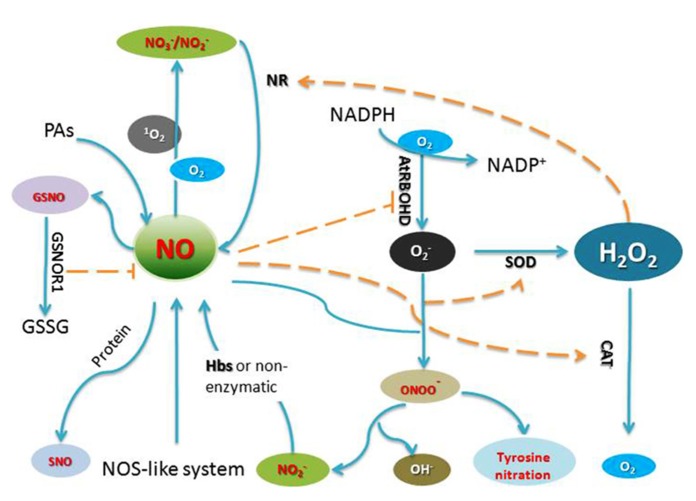
**Generation of and crosstalk by RNS and ROS in plant cells.** AtRBOHD, an NADPH oxidase; GSNO, *S*-nitrosoglutathione; GSNOR1, *S*-nitrosoglutathione reductase 1; GSSG, glutathione disulfide; NR, nitrate reductase; SOD, superoxide dismutase; Hbs, Hemoglobin; PAs, polyamines; CAT, catalase.

Nitric oxide (NO) is a gaseous free radical which was first found to play a crucial role in plant and mediating defense reactions against bacterial pathogens (Noritake et al., 1996; Delledonne et al., 1998). Increasing evidence suggests that NO, as a signal mediator, plays a key role in many physiological and developmental processes, such as germination, leaf expansion, lateral root development, flowering, stomatal closure, crosstalk with plant hormones, defenses against biotic and abiotic stresses (He et al., 2004; Hong et al., 2008; Leitner et al., 2009; Wilkins et al., 2011; Liu et al., 2013; Yadav et al., 2013). In plants, mitochondria and chloroplasts are organelles that are thought to contribute to NO generation *in vivo* (Galatro et al., 2013; Vanlerberghe, 2013). Although a long standing search for an NO synthase (NOS) in plants similar to NOS enzymes found in mammals has thus far been unsuccessful, suppression of NO signaling in the presence of NOS inhibitors has been reported by several groups, indicating the potential existence of a NOS-like enzyme in plants (Tewari et al., 2013; **Figure 1**).

As a free radical, NO could also react with various intracellular/extracellular targets and form a series of molecules, such as NO radicals (NO^-^), nitrosonium ions (NO^+^), peroxynitrite (ONOO^-^), *S*-nitrosothiols (SNOs), higher oxides of nitrogen (NO_x_) and dinitrosyl-iron complexes among others, collectively these NO derivatives are termed reactive nitrogen species (RNS; Di Stasi et al., 2002). The functions of RNS, in plant cells are complex because they are implicated in many different physiological processes. *S*-nitrosylation, the covalent attachment of an NO moiety to a reactive cysteine thiol to form an SNO, has emerged as a prototypic redox-mediated modification in plants. For example, *S*-nitrosylation of methionine adenosyltransferase 1 (MAT1; Lindermayr et al., 2006), the *Arabidopsis* type-II metacaspase AtMC9 (Belenghi et al., 2007), PrxII E, a member of the peroxiredoxin family (Romero-Puertas et al., 2007b), non-expression of pathogenesis-related protein 1 (NPR1; Tada et al., 2008), *Arabidopsis thaliana* salicylic acid (SA) binding protein 3 (AtSABP3; Wang et al., 2009), TGACG motif binding factor 1 (TGA1) family (Lindermayr et al., 2010), nicotinamide adenine dinucleotide phosphate (NADPH) oxidase AtRBOHD (Yun et al., 2011), cytoskeletal proteins (Yemets et al., 2011), auxin receptor-transport inhibitor response 1/auxin signaling F-box (TIR1/AFB; Terrile et al., 2011), glyceraldehyde-3-phosphate dehydrogenase (GAPDH; Lin et al., 2012) and also *Arabidopsis* histidine phosphotransfer protein (AHP1; Feng et al., 2013) have been reported. These data implies that protein *S*-nitrosylation is a key redox-based modification in plants and a pivotal mechanism to convey NO bioactivity. Peroxynitrite (ONOO^-^), formed from ${NO}_{2}^{-}$ and NO, is also capable of reacting with many classes of biomolecules such as antioxidants and proteins, triggers defense responses in animals and plants (Rubbo et al., 1994a,b). In *Arabidopsis*, ONOO^-^ could induce hypersensitive response (HR) and defense-related gene expression (Alamillo and Garcia-Olmedo, 2001). Very recently, protein tyrosine nitration, addition of an nitro group (NO_2_) to one of the two equivalent ortho carbons of the aromatic ring of Tyr residues and metal nitrosylation, was reported as a new important RNS-mediated post-translational modification (Saito et al., 2006; Astier and Lindermayr, 2012; Tanou et al., 2012; Begara-Morales et al., 2013; Chaki et al., 2013). These findings not only deepen our understanding of NO signaling and function in plants, but also indicate the existence of RNS cross-talk with other signaling pathways, such as those orchestrated by auxin, cytokinin, SA, jasmonic acid (JA), ethylene (ET), and reactive oxygen species (ROS).

## REACTIVE OXYGEN SPECIES

Reactive oxygen species including hydrogen peroxide (H_2_O_2_), superoxide anion ${NO}_{2}^{-}$, hydroxyl radicals (.OH) and singlet oxygen (^1^O_2_) have all been implicated in the control of biological processes in plants. Mitochondria as an “energy factory” are believed to be a major site of ROS production. Alternative oxidase (AOX) has an important influence on both ROS and RNS generation by the respiratory chain in mitochondria (Vanlerberghe, 2013). Peroxisomes are subcellular organelles with an essentially oxidative type of metabolism and produce superoxide radicals ${NO}_{2}^{-}$ as a consequence of their normal metabolism. Chloroplasts are also a major site of ROS generation in plants (Hideg et al., 2006). The superoxide radicals ${NO}_{2}^{-}$ and singlet oxygen (^1^O_2_) are produced in chloroplasts by photo-reduction of oxygen and energy transfer from triplet excited chlorophyll to oxygen, respectively (**Figure 1**).

Hydrogen peroxide, a ROS of major biological significance, can form as a result of the reaction of superoxide and also can be generated by specific enzymes (Noctor et al., 2000; Gechev et al., 2006). An oxidative burst, with rapid ${NO}_{2}^{-}$ synthesis and its subsequent dismutation to H_2_O_2_ in the apoplast, is a common response to pathogens, elicitors, wounding, heat, ultra-violet light, and ozone (Orozco-Cardenas et al., 2001; Rao and Davis, 2001). Besides its directly oxidative activity, it is now clear that H_2_O_2_ has a key signaling role in plants (Gechev et al., 2006; Jiang et al., 2011). H_2_O_2 _ can induce gene expression and modulates signaling proteins, such as protein phosphatases (PP), protein kinases (PK), transcription factors and calcium channels that are located in the plasma membrane or elsewhere (Neill et al., 2002; Lin et al., 2012).

## ROS AND NO SIGNALING IN THE HYPERSENSITIVE RESPONSE

**FIGURE 2:**
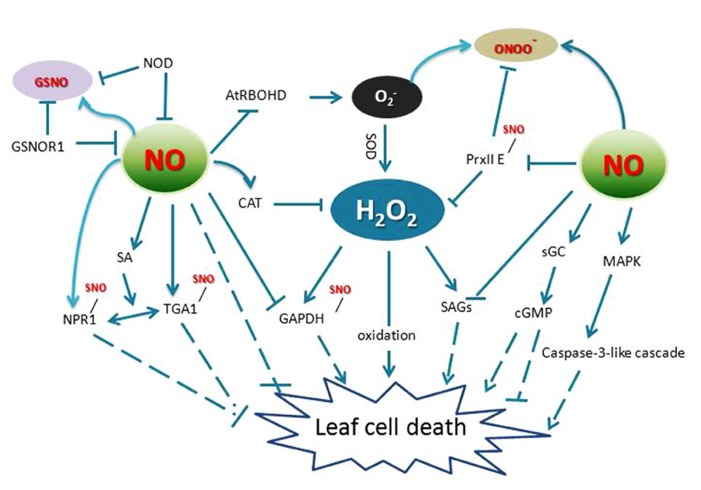
**Crosstalk of RNS and ROS in leaf cell death.** AtRBOHD, an NADPH oxidase; GAPDH, glyceraldehyde 3-phosphate dehydrogenase; GSNO, *S*-nitrosoglutathione; GSNOR1, *S*-nitrosoglutathione reductase 1; NPR1, non-expression of pathogenesis related protein 1; TGA1, TGACG motif binding factor 1; NR, nitrate reductase; SAG, senescence-associated genes; PrxII E, peroxiredoxin II E; NOD, NO degrading dioxygenase; sGC, soluble guanylate cyclase. MAPK, mitogen-activated protein kinase; SOD, superoxide dismutase; CAT, catalase; cGMP, cyclic guanosine monophosphate; sGC, soluble guanylate cyclase.

A well-documented form of plant programed cell death (PCD) is the HR, characterized by the rapid cell death surrounding infection sites. The HR shows some similarity to the characteristics of animal apoptosis, such as membrane dysfunction, vacuolization of the cytoplasm, chromatin condensation, and endonucleolytic cleavage of DNA (Greenberg and Yao, 2004; Choi et al., 2013; Iakimova et al., 2013). Both NO and ROS have been implicated in controlling the HR process. One of the key determinants for the HR is the balance between intracellular NO and ROS levels (Delledonne et al., 2001; Zaninotto et al., 2006). Following pathogen recognition, NO accumulation occurs concomitant with an oxidative burst, which consists of a biphasic production of apoplastic ROS at the site of attempted invasion (Romero-Puertas et al., 2004). In this context, NO and H_2_O_2_ are thought to function in combination to promote HR cell death. For example, either of them could cause the release of cytochrome *c* from mitochondria, and affect the caspase-like signaling cascade, leading to the HR (Mur et al., 2006; Tan et al., 2013). Some key components of the defense signaling cascade that are known to be affected by ROS and NO activity include mitogen-activated protein kinases (MAPKs) and phosphatases (**Figure 2**). Thus, modulation of a central MAPK cascade may converge both H_2_O_2_ and NO signaling pathways activated in response to pathogen infection. In tomato cell suspensions, upon xylanase perception, cells activate a protein kinase pathway required for NO formation and *S*-nitrosylation-dependent mechanisms which are involved in downstream signaling, leading to production of polyamine and ROS production (Lanteri et al., 2011).

Interestingly, many proteins are targets of both NO and H_2_O_2_ (**Figure 2**). For example, GAPDH that plays a role in mediating ROS signaling in plants is a direct target of H_2_O_2_ and it is also a target of NO-mediated *S*-nitrosylation, which blunts its activity (Lindermayr et al., 2005). Also, MAT in mammals is inactivated by H_2_O_2_ through a reversible and covalent oxidation of a Cys residue. The same Cys residue is also a target for NO, which similarly causes enzyme inactivation (Hancock et al., 2005). Further, PrxII E not only reduces H_2_O_2_ and alkyl hydroperoxides (Dietz, 2003a,b; Horling et al., 2003), but also functions in detoxifying peroxynitrite. *S*-nitrosylation of PrxII E during the defense response regulates the antioxidant function of this key enzyme and might contribute to the HR (Romero-Puertas et al., 2007a,b; **Figure 2**). As a useful tool to elicit ROS-activated responses, ozone (O_3_) has been shown to induce HR-like cell death. During this process, NO accumulation preceded accumulation of ET, JA, SA, and leaf injury, implies that NO is an important signaling molecule in response to O_3_ exposure (Rao and Davis, 2001; Ahlfors et al., 2009).

Contrary to its program cell death functions in the HR, NO can also scavenge H_2_O_2_ and protects plant cells from damage under certain circumstances (Beligni et al., 2002; Crawford and Guo, 2005). NO donors affect both wounding-induced H_2_O_2_ synthesis and wounding- or JA-induced expression of defense genes (Grun et al., 2006). In *Arabidopsis*, *S*-nitrosoglutathione reductase 1 (GSNOR1) is a key regulator that indirectly controls the global levels of protein *S*-nitrosylation (SNO). Loss-of-function mutations in *GSNOR1 *increased total cellular NO and SNO content and compromised both non-host and resistance (*R*) gene-mediated protection and also disabled basal defense responses (Feechan et al., 2005; Wang et al., 2010; **Figure 2**). Further, the mutant *atgsnor1–3* was also perturbed in thermotolerance and resistance to paraquat (1,1^′^-dimethyl-4,4^′^-bipyridinium dichloride), which induces the production of superoxide and H_2_O_2_ in wild type leaves (Lee et al., 2008; Chen et al., 2009). Consistent with these results, wild-type plants treated with an NO donor displayed resistance to paraquat (Chen et al., 2009). These studies showed that the *Arabidopsis* *GSNOR1/HOT5/PAR-2* gene not only regulates SA signaling and thermotolerance by modulating the intracellular SNO level, but also acts downstream of superoxide to regulate cell death.

Interestingly, the increased levels of SNOs in *atgsnor1–3* plants potentiated the HR even in the absence of the cell death agonist SA and apoplastic ROS synthesis. Surprisingly, NO *S*-nitrosylates the NADPH oxidase, AtRBOHD, at Cys890, diminishes its ability to synthesize ROS. This cysteine is also evolutionarily conserved and specifically *S*-nitrosylated in both human and fly NADPH oxidases, suggesting that this mechanism may govern immune responses in both plants and animals (Yun et al., 2011). Thus, NO may control ROS production through protein *S*-nitrosylation to further control the development of cell death processes. Collectively, these findings have provided significant insights into the understanding of the mechanisms underpinning ROS and RNS function in plants, revealing that the ROS/RNS pathway in plant PCD is highly complex and is mediated at least in part by crosstalk with several phytohormone signaling networks.

## NO AND ROS CROSSTALK IN LEAF SENESCENCE

Leaf senescence, thought to be another form of plant PCD, is the final stage of leaf development, which is not only controlled by organ age but also triggered by adverse environmental factors (Pourtau et al., 2004; Munns, 2005; Masclaux-Daubresse et al., 2007; Jing et al., 2008; Wu et al., 2012). Additionally, phytohormones such as ET, SA, JA, auxin, ABA, and cytokinins all affect leaf senescence (Lim et al., 2007). In *Arabidopsis*, the level of H_2_O_2_ increases dramatically in leaf tissue during senescence. In addition to its role in oxidizing macromolecules such as proteins and lipids, H_2_O_2_ has also been proposed to function as a signal to induce the expression of genes involved in the senescence process (Cui et al., 2013). In agreement with its lower antioxidant capacity, senescent leaf tissue was found to contain elevated levels of ROS. In this context, a number of senescence-associated genes (SAGs) characterized from *Arabidopsis* could be induced by ozone (Miller et al., 1999) and the expression of many other SAGs were also induced by ROS (Navabpour et al., 2003), indicating that ROS might function as a signal to promote senescence. Interestingly, senescence-associated NAC genes (senNACs), key regulators of leaf senescence, were also found to be rapidly and strongly induced by H_2_O_2_ treatment in both leaves and roots (Balazadeh et al., 2010, 2011). Thus, ROS has a dual role in leaf senescence: to promote the cell death process by directly oxidizing target macromolecules and to drive the expression of senescence-related genes.

Distinct from the positive role of ROS in senescence, NO can both provoke and impede this process, dependent upon its concentration and subcellular location. NO may alleviate the toxicity of ROS and has thus acted as a leaf senescence delaying factor in plants. The NO-deficient mutant *nos1/noa1* showed early leaf senescence (Niu and Guo, 2012) and similarly *Arabidopsis* expressing an NO degrading dioxygenase (NOD) displayed a senescence-like phenotype (Mishina et al., 2007; **Figure 2**). Furthermore, the level of NO is related with the senescence process and is thought to be an essential component involved in plant senescence signaling cascades. In *Arabidopsis* mutant *dnd1*, which lacks a plasma membrane-localized cation channel (CNGC2), early senescence-associated phenotypes (such as loss of chlorophyll, expression level of senescence associated genes, H_2_O_2_ generation, lipid peroxidation, tissue necrosis, and SA levels) were all elevated relative to wild type. Basal levels of NO in *dnd1* leaves were lower than wild type, suggesting that the function of CNGC2 may impact downstream “basal” NO production in addition to its role linked to NO signaling (Ma et al., 2010). NO generation is therefore thought to act as a negative regulator during plant leaf senescence signaling. The protective effect of NO against ROS induced cell death can also be linked to the enhanced activity of antioxidant enzymes, as negative regulator of the chlorophyll catabolic pathway and as drivers for positively maintaining the stability of thylakoid membranes during leaf senescence (Liu and Guo, 2013).

On the other hand, NO can also promote the leaf senescence. *Arabidopsis* AtFer1, one of the best characterized plant ferritin isoforms to date, strongly accumulates upon treatment with excess iron, via an NO-mediated pathway. The AtFer1 isoform is functionally involved in events leading to the onset of age-dependent senescence in *Arabidopsis* and its iron-detoxification function during senescence is required when ROS accumulates (Murgia et al., 2007). Recently identification of an NO accrual mutant *noe1* (*nitric oxide excess 1*) in rice revealed that *NOE1* encoded a rice catalase (CAT) OsCATC. Interestingly, *noe1* plants exhibited an increase of H_2_O_2_ in their leaves, which consequently promoted NO production via activation of nitrate reductase. Removal of excess NO reduced cell death in both leaves and suspension cultures derived from *noe1* plants, implicating that NO acts as an important endogenous mediator of H_2_O_2_-induced leaf cell death. Reduction of intracellular SNO levels, generated by over-expression of OsGSNOR alleviated leaf cell death in *noe1* plants. Thus, *S*-nitrosylation was also involved in light-dependent leaf cell death in *noe1*. Collectively, these data suggest that both NO and SNOs are important mediators in the process of H_2_O_2_-induced leaf cell death in rice (Lin et al., 2012; Wang et al., 2013). OsGSNOR**in *noe1 *plants reduced SNO levels, consistent with a key role for this enzyme in SNO homeostasis. Moreover, the results show that no change in H_2_O_2_ content occurred in either *GSNOR*-overexpressing or *GSNOR*-RNAi transgenic lines**in the context of *noe1 *background, suggesting that NO might function downstream of H_2_O_2_ in a light-driven leaf cell death in rice. It was found that NO treatment led to rapid cell death and induced H_2_O_2_ accumulation in maize leaves, and pharmacological studies also suggested that NO-induced cell death is in part mediated via H_2_O_2_, therefore H_2_O_2_ may be involved in NO-induced cell death in maize leaves (Kong et al., 2013). These discrepancies for the role of NO in cell death might be due to the differences in plant species, redox state, and growth conditions. Both NO and H_2_O_2_ could induce leaf cell death during which they could crosstalk with each other through different pathways.

## NO AND ROS IN OTHER TYPES OF PLANT CELL DEATH

Some reports also describe the cross-talk of NO and ROS in other kinds of cell death in plants. Gibberellin (GA)-induced PCD in barley (*Hordeum vulgare* cv.**Himalaya) aleurone layers is mediated by ROS and NO is a protective antioxidant. NO donors delay this PCD process, but do not inhibit metabolism in general, or the GA-induced synthesis and secretion of alpha-amylase. The amounts of CAT and superoxide dismutase (SOD) are greatly reduced in aleurone layers treated with GA. Treatment with GA in the presence of NO donors delays the loss of CAT and SOD. Thus, NO may be an endogenous modulator of PCD in barley aleurone cells (Beligni et al., 2002). Furthermore, the exogenous application of NO rendered the plants more tolerant to arsenic (As)-induced oxidative damage by enhancing their antioxidant defense and glyoxalase system (Hasanuzzaman and Fujita, 2013). Previous work has also shown that NO acts as a pivotal positive mediator in cadmium (Cd)-induced PCD in suspension cell cultures. NO strongly counteracts Cd-induced ROS mediated cytotoxicity in *Brassica juncea* by controlling antioxidant metabolism (De Michele et al., 2009; Verma et al., 2013). Similarly, a role for NO as an antioxidant during heavy metal mediated toxicity has been highlighted recently by Saxena and Shekhawat (2013).

On the other hand, NO could also aid ROS-induced PCD. In pollen-pistil interactions, self-incompatibility (SI) induces relatively rapid and transient increases in ROS and NO. As ROS/NO scavengers alleviated both the formation of SI-induced actin punctate foci and also activation of a DEVDase/caspase-3-like activity (Wilkins et al., 2011). In tobacco BY-2 cells, sphinganine or dihydrosphingosine (d18:0, DHS) induce a calcium dependent PCD and trigger H_2_O_2_ production via the activation of NADPH oxidase(s). They also promote NO production, which is required for cell death induction (Da Silva et al., 2011). NO accumulated in Cd-induced PCD and promoted Cd-induced *Arabidopsis* PCD by promoting MPK6-mediated caspase-3-like activation (Ye et al., 2013). So the different roles of RNS in PCD and their crosstalk with ROS depend on the plant species, growth conditions and redox status.

## CONCLUSION

In plants, RNS and ROS synthesis is a routine requirement for cells to undergo PCD, these small molecules can act either synergistically or independently (Clarke et al., 2000; Orozco-Cardenas and Ryan, 2002; Bright et al., 2006). The accumulating data suggests significant cross-talk occurs between RNS and ROS (**Figure 1**), although the clear relationship of RNS and ROS in the process of cell death remains elusive. NO and ROS could regulate the synthesis each other. During HR, NO can affect ROS synthesis through *S*-nitrosylating NADPH oxidase AtRBOHD (Yun et al., 2011). On the other hand, in rice *noe1 *mutant, in the absence of OsNOE1/OsCATC function, the accumulation of H_2_O_2_ induces NO production through elevating nitrate reductase expression, which is further integral to H_2_O_2_ induced leaf cell death through *S*-nitrosylation of GAPDH and thioredoxin (Lin et al., 2012; Wang et al., 2013). Cross-talk of NO and H_2_O_2_ is a prominent feature in the activities of these small molecules. RNS and ROS also play important roles in modulating the activity of target proteins. A complete list of signaling pathways regulated by ROS or RNS still awaits identification, the data presented in this review are therefore far from offering a comprehensive picture of the function of NO and ROS during plant PCD. Thus, further work is needed to understand how these key molecules trigger the onset and development of plant cell death.

## Conflict of Interest Statement

The authors declare that the research was conducted in the absence of any commercial or financial relationships that could be construed as a potential conflict of interest.
